# Where we are now – Monitoring health and disability in older European populations and what harmonisation efforts (CLESA, EPOSA) could tell

**DOI:** 10.1186/1753-6561-7-S4-K1

**Published:** 2013-08-23

**Authors:** Dorly J H Deeg

**Affiliations:** 1 Department of Epidemiology and Biostatistics and the EMGO Institute for Health and Care Research, VU University Medical Center, Amsterdam, the Netherlands

## 

In all countries of Europe, the population is ageing. This shift involves information needs on a variety of ageing-related themes. For better insight into similarities and differences among European countries, there is an increasing platform to support the added value of comparative analyses on ageing across the European Union. As many survey data on ageing exist, the most fruitful way forward is to share datasets and to harmonise concepts, indicators, and methods as much as possible. However, harmonisation may involve various difficulties. Two examples are given.

In the context of the EU fifth Framework Programme, the Comparison of Longitudinal European Studies on Ageing (CLESA, 2001-2004) project was among the first projects to attempt harmonisation of data on health and quality of life. Post-harmonisation was undertaken using population-based datasets in six countries. An example is given of the harmonisation process of the concept of ADL disability, which allowed the comparison of disability-free life expectancy across the six countries. A North-South gradient was found, showing shorter disability-free life expectancies in Spain, Italy and Israel as compared to Finland, Sweden and the Netherlands. This gradient was suggested to be caused by differences in educational level and in family culture.

The European Project on Osteoarthritis (EPOSA, 2009-2013) (http://www.eposa.org) on the personal and societal consequences of osteoarthritis in older people comprises a more recent attempt at harmonisation, using population-based cohort studies in six countries. Here, post-harmonisation was less successful in that the main variable, osteoarthritis (OA), was defined in too different ways. The heterogeneity of OA definitions hampers comparing prevalence rates, and possibly, associations of OA with quality of life. Therefore, in a follow-up project, new data collection in the six cohorts was carried out, with pre-harmonisation of measurement instruments, including a standardised clinical assessment of OA. Preliminary findings show differences in OA prevalence, with higher rates in Southern Europe (Italy and Spain) than in middle and northern Europe.

In conclusion, post-harmonisation may be a cost-effective approach to use existing data, but may not always lead to comparable data. Even in case of pre-harmonisation, for any findings from multi-country studies, it should be considered to what extent differences observed are still linked to differences in data collection or are indicative of real cross-national differences.

